# Synergistic interaction of eugenol and antimicrobial drugs in eradication of single and mixed biofilms of *Candida albicans* and *Streptococcus mutans*

**DOI:** 10.1186/s13568-020-01123-2

**Published:** 2020-10-19

**Authors:** Huma Jafri, Gopa Banerjee, Mohd Sajjad Ahmad Khan, Iqbal Ahmad, Hussein Hasan Abulreesh, Abdullah Safar Althubiani

**Affiliations:** 1 Department of Agricultural Microbiology, Faculty of Agricultural Sciences, Aligarh Muslim University, Aligarh, 202002 India; 2 Department of Microbiology, King George Medical University, Lucknow, 226020 India; 3 Department of Basic Sciences, Deanship of Preparatory Year and Supporting Studies, Imam Abdulrahman Bin Faisal University, P.O. Box 1982, Dammam, 34212 Saudi Arabia; 4 Department of Biology, Faculty of Applied Science, Umm Al-Qura University, Makkah, Kingdom of Saudi Arabia

**Keywords:** *C. albicans*, *S. mutans*, SMIC, PMIC, Biofilm eradication, FICI, Synergy

## Abstract

In vitro eradication of the *C. albicans* and *S. mutans* mixed biofilms by eugenol alone and in combination with the antimicrobial drugs. Previously characterized strains of *C. albicans* (CAJ-01 and CAJ-12) and *S. mutans* MTCC497 were used to evaluate the eradication of biofilms using XTT reduction assay, viability assay, time dependent killing assay and scanning electron microscopy (SEM). Synergistic interaction was assessed by checkerboard method. Sessile MIC (SMIC) of eugenol was equivalent to the planktonic MIC (PMIC) against *C. albicans* and *S. mutans* mixed biofilms. SMIC of fluconazole and azithromycin was increased upto 1000-folds over PMIC. Eradication of single or mixed biofilms was evident from the viability assay and SEM. At 1 × MIC of eugenol, log_10_CFU count of *C. albicans* cells were decreased from 6.3 to 4.2 and 3.8 (*p* < 0.05) in single and mixed biofilms, respectively. SEM studies revealed the eradication of *C. albicans* and *S. mutans* cells from glass surface at 800 µg/mL concentration of eugenol. Time dependent killing assay showed dose dependent effect of eugenol on pre-formed CAJ-01, CAJ-12 and *S. mutans* biofilm cells. Eugenol was highly synergistic with fluconazole (FICI = 0.156) against CAJ-12 single biofilms. However, the combination of eugenol and azithromycin showed maximum synergy (FICI = 0.140) against pre-formed *C. albicans* and *S. mutans* mixed biofilms. These findings highlighted the promising efficacy of eugenol in the eradication of biofilms of two oral pathogens (*C. albicans* and *S. mutans*) in vitro and could also be exploited in synergy with fluconazole and azithromycin in controlling oral infections.

## Key points

Mixed biofilm formation by the *C. albicans* and *S. mutans*, eradication of mixed biofilms by combine treatment of essential oil and antimicrobial drugs.

## Introduction

A variety of microbial species can colonize and adhere to oral cavity and cause number of infectious diseases including dental infections (Li et al. 2000; Zhu et al. 2018). The members of oral microflora can form biofilms both on tissues or solid surface (dental implants). They interact among themselves and can develop mixed biofilms, and thereby, increasing the pathogenesis of disease. In industrialized countries, problem of dental caries has increased at an alarming rate in children and adults. About 60–80% of the children have been suffering from dental caries (Maripandi et al. 2011). Dental plaque is the most classic example of pathogenic biofilms (Vasudevan 2017). *C. albicans* can cause severe infection along with *S. mutans* in plaque biofilms in children with early childhood caries (Falsetta et al. 2014; Hwang et al. 2017). Approximately 25.5% of healthy individuals were encountered with both *C. albicans* and *S. mutans* in the denture plaque (Sztajer et al. 2014). The accumulation of *S. mutans* triggers the *C. albicans* biofilm formation under in vitro conditions (Barbosa et al. 2016) and may satisfy the requirement of metabolites or growth stimulating factors in mixed biofilm conditions. *Streptococci* also lead to the production of the cell wall anchored protein that assists in binding of *Candida* cells. *C. albicans* further utilizes the metabolized products and stimulate the production of ample amount of EPS which is important for aggregation and accumulation of *S. mutans* cells to develop mixed biofilms (Falsetta et al. 2014). Additionally, the increased population of *Candida* cells may also reduce the diversity of oral microbiome and substitute the microbial community with *Strepoccocci* (Kraneveld et al. 2012). The synergistic interaction between these pathogens helps in the establishment and pathogenicity in the oral environment (Morales and Hogan 2010).

Eradication of these pathogens is not always easy and successful because of their strong and compatible biofilm forming ability. It is evident from several studies that microorganisms in biofilm mode are less susceptible to the traditionally used antimicrobial drugs compared to the planktonic mode (Algburi et al. 2017). *C. albicans* has shown resistance against azole drugs and extended to echinocandins as well (Pristov and Ghannoum 2019). Also, the frequency of drug resistance in bacteria has been increased over the past decade. Various factors affect the susceptibility of the pathogens enfolded in a biofilm such as activation of biofilm phenotype, stress responses and decrease in the penetration of antimicrobial agents due to the EPS matrix (Mah Thien-Fah and O'Toole George 2001). Alternative strategies or development of more efficient antimicrobial agents showing activity against pathogenic biofilms are of great practical significance. The increasing incidence of multi drug resistance (MDR) in microbial pathogens and slow progress in novel anti-infective drug discovery has necessitated to scrutinize the traditionally used medicinal and herbal plants as an alternative drug (Khan et al. 2012; Pan et al. 2013; Yuan et al. 2016; Cheesman et al. 2017). Nowadays, there has been increasing interest in exploring plant materials as a source of new agents for the development of therapeutic compounds due to their diversity in bioactive compounds and safe use in traditional system of medicine. This is one of the potential approaches for the treatment of infections as they are safe for human and animal health. Essential oil compounds exhibit anti-biofilm properties, and these characteristics have been studied (Jafri et al. 2019). Eugenol is a major active compound of *S. aromaticum* and with demonstrated antimicrobial action. The mechanism of action of eugenol is based on its ability to interfere cell wall and cytoplasmic membrane synthesis leading to leakage of intracellular material. Recently, eugenol has been reported to inhibit single and mixed biofilms of *C. albicans* and *S. mutans* biofilms (Jafri et al. 2019). Many authors have reported synergistic interaction between essential oil compounds with antimicrobial drugs as possible strategy to combat single and mixed biofilm infections (Khan et al. 2012; Borges et al. 2016; Fernandes et al. 2016; Roy et al. 2018). Therefore, we hypothesize that eugenol with multi-target activity might be useful in controlling mixed biofilms alone or in combination with fluconazole and or azithromycin. In this study, an in vitro eradication of the *C. albicans* and *S. mutans* mixed biofilm by eugenol alone and in combination with the antimicrobial drugs was studied using previously characterized strains of *C. albicans* and *S. mutans* MTCC497.

## Materials and methods

### Microbial pathogens and microbiological media

As previously identified and characterized, microbial strains of *C. albicans* (CAJ-01 and CAJ-12) and *S. mutans* MTCC497 were selected on the basis of strong biofilm forming ability (Jafri et al. 2019). The strains of *C. albicans* and *S. mutans* were maintained on Sabouraud dextrose agar (SDA) and nutrient agar (NA) slants at 4 °C, respectively. Sabouraud dextrose broth/agar (SDB/SDA), nutrient agar, brain heart infusion broth (BHIB) and tryptic soy broth (TSB), were obtained from Hi-Media Laboratory, Mumbai, India. RPMI 1640 medium was purchased from Sigma, New Delhi, India. CAJ-01 strain was deposited to MTCC, Chandigarh, India with collection number MTCC, 13,013.

### Antimicrobial drugs and eugenol

Drug powders of fluconazole (Pfizer Co., India), amphotericin B (Hi-Media, India), azithromycin (Cipla, Mumbai, India) and chloramphenicol (Cipla, Mumbai, India) were used in this study. Stock solutions of antifungal drugs were prepared in dimethyl sulphoxide (DMSO) whereas antibacterial drugs in distilled water at a concentration of 25 mg/mL and stored at 4 °C for not more than one week. Eugenol (99% purity) was purchased from Hi-Media Laboratory, Mumbai, India. DMSO (1%) was used to dilute eugenol.

### Determination of planktonic minimum inhibitory concentration (PMIC) of test agents against mixed *C. albicans* and *S. mutans* cells

Susceptibility of planktonic cells of *Candida* and *Streptococcus* under mixed conditions was evaluated against the test agents using a modified method as described by Li et al. (2015). Briefly, 100 µL of prepared two-fold dilution of test agents (eugenol, fluconazole and azithromycin), 50 µL of *Candida* (2 × 10^3^ CFU/mL) and 50 µL of *S. mutans* inoculum (2 × 10^5^ CFU/mL) were added to the 96-well microtiter plate. Plates were incubated for 48 h at 37 °C. A well without any antimicrobial agent considered as negative control. MIC was calculated as the lowest concentration of antimicrobial agent that inhibited the visible growth of test organisms. Each experiment was conducted two times in triplicate.

### Determination of sessile minimum inhibitory concentration (SMIC) of test agents against mixed *C. albicans* and *S. mutans* cells

Mixed biofilms of *C. albicans* (CAJ-01 and CAJ-12) and *S. mutans* MTCC497 was allowed to develop as described in our previous study (Jafri et al. 2019). Once the biofilms formed, non-adherent cells were washed thrice with sterile phosphate buffer saline (PBS). Next, 100 µL of prepared two-fold serial dilutions of fluconazole, amphotericin B and eugenol in RPMI 1640 medium (for *Candida* biofilms), azithromycin and eugenol in BHI medium (for bacterial biofilms) and fluconazole, azithromycin and eugenol in TSB medium (for mixed biofilms) were added to each biofilm well of microtiter plates and incubated at 37℃ for 48 h. SMIC of the test compounds were assessed using XTT reduction assay. Sessile MIC (SMIC) of test agents was considered as the concentration eradicating 80% of pre-formed biofilm cells (Khan et al. 2012).

### Determination of viability of the single and mixed *C. albicans* and *S. mutans* biofilm cells

Single and mixed biofilms of *C. albicans* (CAJ-01) and *S. mutans* MTCC497 was allowed to develop in 96-well plate (Jafri et al. 2019). Further, the viability of biofilm cells was determined by slightly modified method of Budzyńska et al. (2017). After incubation, biofilm mass were scraped off the walls of the wells by using a sterile scalpel and 100 µL of PBS was added into the wells. The resulting suspension containing the biofilm cells was sonicated for 5 min to disturb the aggregates. After that cell suspension was serially diluted and spread on SDA/NA plate. For mixed biofilms, cell suspension was spread on SDA plate supplemented with chloramphenicol (for *C. albicans*) and BHI agar plate supplemented with amphotericin B (for *S. mutans*). The resulting CFU count of biofim cells were calculated after 24 h incubation at 37 °C. Each assay was conducted two times in triplicate and mean log CFU was used to determine the viability of cells.

### Scanning electron microscopy of pre-formed single and mixed biofilm cells treated with eugenol

Single and mixed biofilms were allowed to form using 24-well flat bottom culture plate with sterile coverslips using method as described by Harriott and Noverr (2009). Pre-formed single and mixed biofilms of CAJ-01 and *S. mutans* MTCC497 cells were treated by adding 1000 µL of prepared dilution of eugenol in the plate wells. The plates were incubated at 37 °C for 48 h. Glass coverslips containing biofilm cells were washed with PBS and fixed with 5% glutaraldehyde in cocodylate buffer in a graded concentration of ethanol (25, 50, 75, 95 and 100%), immersed in hexamethyldisilazane and dried overnight at room temperature. The glass coverslips were then mounted on aluminium stubs with silver paint and sputter coated with gold using scanning electron microscope (JSM 6510, LV, JEOL, JAPAN). The micrographs were taken and processed.

### Kinetics of killing of pre-formed *C. albicans* and *S. mutans* biofilm cells

To determine the potency of eugenol and antimicrobial drugs (fluconazole, amphotericin B and azithromycin), time kill assay of *C. albicans* and *S. mutans* biofilm cells were performed using method as described and modified by Khan and Ahmad (2012) and Yadav et al. (2015) respectively. Pre-formed *Candida* and *S. mutans* biofilm cells were treated with 2× MIC of eugenol and antimicrobial drugs. Samples of *Candida* and *S. mutans* biofilms were removed immediately, serially diluted and plated on SDA and NA plate respectively. For *Candida* biofilms, the treated biofilm cells were collected after 2, 4, 8, 12, 24 and 48 h of incubation whereas treated bacterial biofilms cells were collected after incubation of 0.5, 1, 1.5, 3, 6, 12, 24 and 48 h. After completion of incubation period, the wells were washed to remove loosely adhered cells and biofilm mass was scraped off the well using a sterile scalpel. The samples were diluted in saline solution and plated on agar plates. Biofilm wells without any treatment served as a control. The mean CFU count was used to determine the viable cells. All the experiments were performed in triplicates in three independent experiments.

### In vitro studies of synergy between eugenol and antimicrobial drugs against single and mixed biofilms of *C. albicans* and *S. mutans*

The combine effect of eugenol and antimicrobial drugs (fluconazole, amphotericin B and azithromycin) on single and mixed biofilm cells were determined by checkerboard microtiter assay as described by Vitale et al. 2005 with little modifications, Briefly, single and mixed biofilms of *C. albicans* and *S. mutans* were allowed to form in 96-well microtiter plate by using the method as described in previous section. Then, pre-formed single and mixed *C. albicans* (CAJ-01 and CAJ-12) and *S. mutans* MTCC497 biofilm cells were treated with various combinations of test agents (eugenol and antimicrobial drugs) by adding 50 µL of each prepared dilution of eugenol and drugs in the vertical and horizontal direction of the plate respectively. Further, the plates were incubated at 37 °C for 48 h. The extent of synergy was determined in terms of FICI index. An FICI index (FICI) was calculated by adding both FICIs. The FICI result was interpreted as: synergistic: FICI ≤ 0.5, no interaction or indifferent > 0.5–4.0, antagonistic > 4.0 (Khan and Ahmad 2012).

## Results

Eugenol showed no increase in SMIC against the mixed biofilm of *C. albicans* plus *S. mutans* MTCC497. Antimicrobial drugs (fluconazole and azithromycin) showed up to 1000-fold increase in SMIC compared to the PMIC against the test microbial strains combination as revealed from Table 1.

**Table 1:** PMIC and SMIC of eugenol and antimicrobial drugs against the mixed cells of *C. albicans* and *S. mutans* MTCC497

Strains	Eugenol		Fluconazole		Azithromycin	
	PMIC	SMIC	PMIC	SMIC	PMIC	SMIC
CAJ-01 plus *S. mutans* MTCC497	800	800	8	1024	128	1024
CAJ-12 plus *S. mutans* MTCC497	200	200	1024	2048	128	1024

Values of PMIC and SMIC are given in µg/mL

**Fig. 1:**
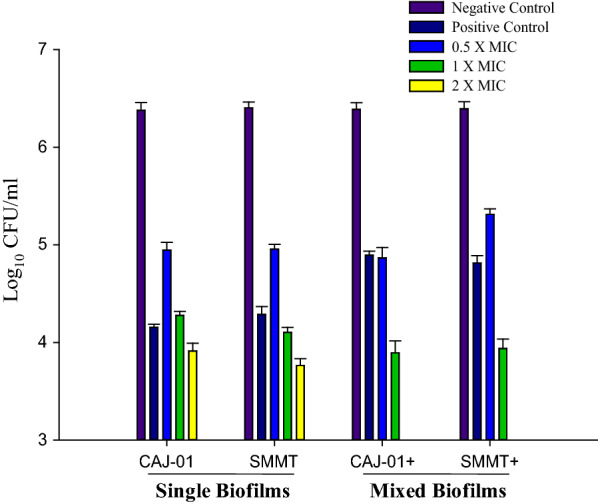
Viability of pre-formed CAJ-01 and *S. mutans* MTCC497 single and mixed biofilm cells on exposure with different concentration of the eugenol. Negative Control: Untreated single and mixed biofilm cells. Positive Control: CHX (200 µg/mL) treated cells, CAJ-01+: Viable cells of CAJ-01 in mixed biofilms, SMMT+: Viable cells of *S. mutans* MTCC497 in mixed biofilms

Further, viability of *C. albicans* and *S. mutans* pre-formed single and mixed biofilm cells was recorded after challenging with the different concentrations of eugenol (0.5 × MIC, 1 × MIC and 2 × MIC) and as depicted in Fig. 1. The data revealed the marked reduction in log_10_CFU count of *C. albicans* and *S. mutans* in single biofilms with the increasing concentration of eugenol. The eradication of single and mixed biofilms was recorded at 1 × MIC and 2 × MIC, which was equivalent to the SMIC of the eugenol. Remarkable reduction in *Candida* and *S. mutans* CFU count was observed at 2× MIC of eugenol in single biofilms. Log_10_CFU count of *C. albicans* cells was reduced from 6.3 to 4.2 and 3.8 (p < 0.05) in single and mixed biofilms, respectively. There were remarkable reduction in CFU count of *C. albicans* cells compared to the *S. mutans* cells at 1× MIC of eugenol in mixed conditions. Chlorhexidine digluconate (CHX) was used as a positive control (200 µg/mL) for biofilm eradication which was a recorded as 50× MIC of CHX against CAJ-01 (Jafri et al. 2019). The activity of eugenol was found comparable to positive control.

**Fig. 2:**
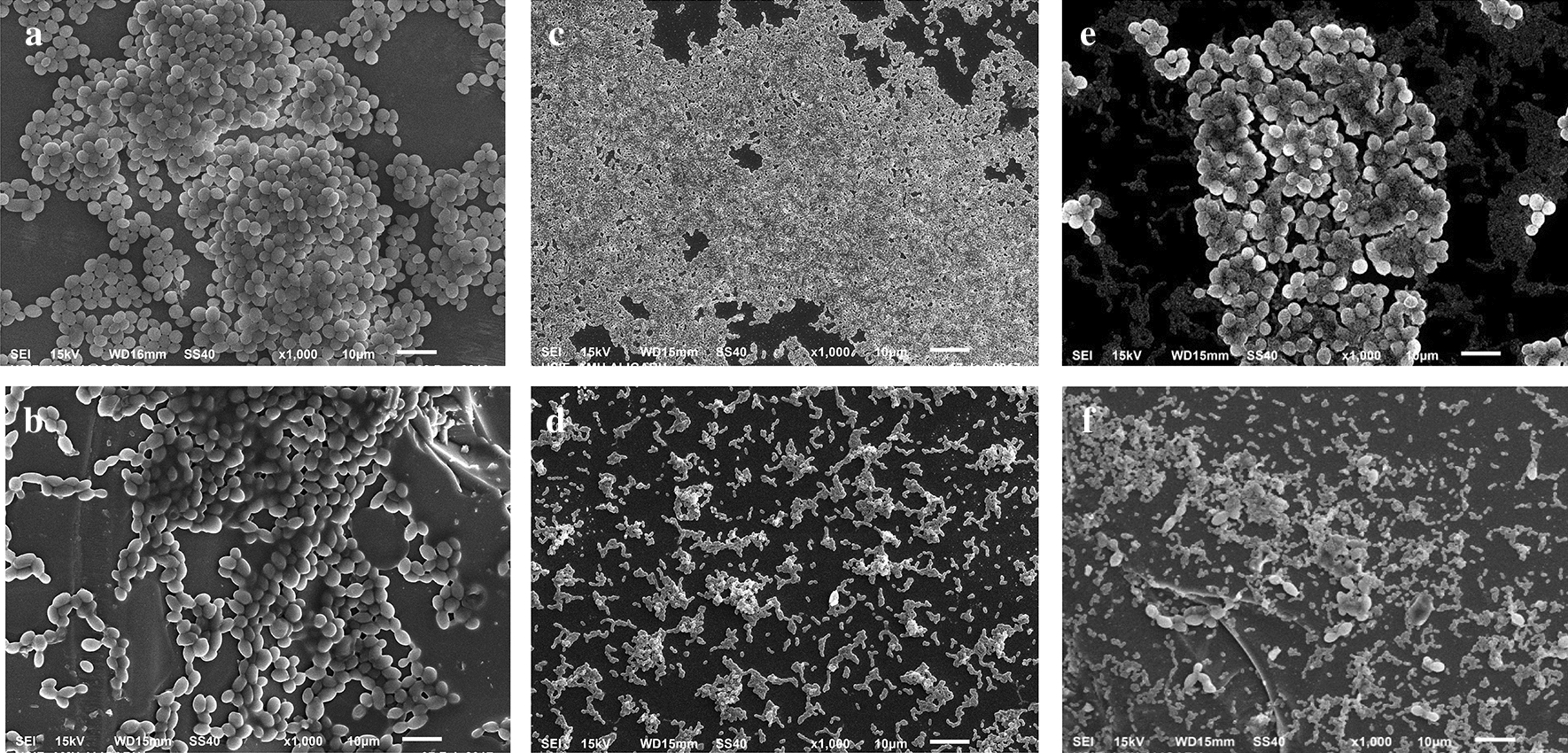
Eradication of pre-formed *C. albicans* (CAJ-01) and *S. mutans* MTCC497 single and mixed biofilms by the treatment of eugenol. **a** CAJ-01 untreated biofilms, **b** Treated with eugenol at 400 µg/mL, **c** *S. mutans* MTCC497 untreated biofilm cells, **d** *S. mutans* MTCC497 biofilm cells treated with eugenol at 400 µg/mL, **e** Untreated CAJ-01 and *S. mutans* MTCC497 mixed biofilm cells (Jafri et al. 2019), **f**. CAJ-01 and *S. mutans* MTCC497 biofilm cells treated with eugenol at 800 µg/mL

Eradication of pre-formed single and mixed biofilms cells was also observed at higher concentration of the test agents (2× MIC) under scanning electron microscope (Fig. 2b). SEM images clearly depicted distortion in *C. albicans* biofilm architecture at 2× MIC of eugenol compared to the untreated control cells. Similarly, pre-formed *S. mutans* biofilms was eradicated at 2× MIC of the test agents. *S. mutans* single species biofilm cells showed abnormal cell structure, lesser number of cell aggregation and removal of EPS matrix (Fig. 2d). Damaged cell morphology, shrinkage of the yeast cells and eradication of *C. albicans* and *S. mutans* cells were also observed in the *C. albicans* (CAJ-01) plus *S. mutans* MTCC497 mixed biofilms after treatment with eugenol (Fig. 2f).

**Fig. 3:**
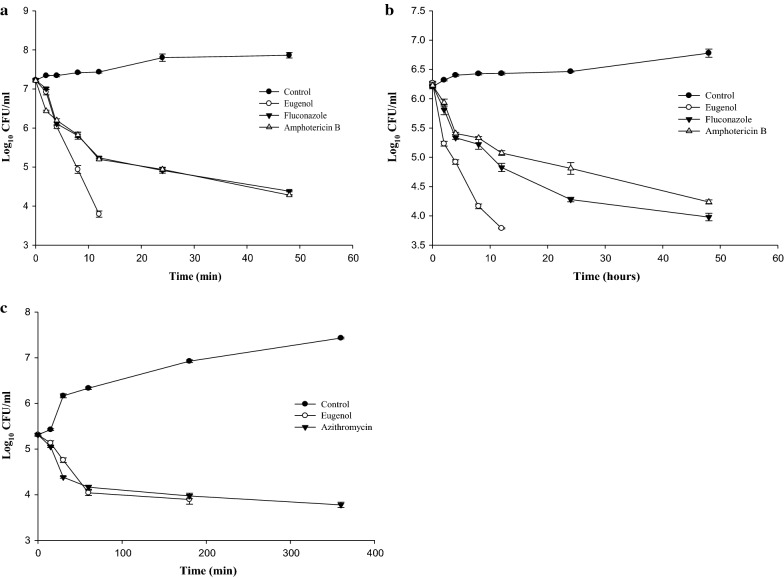
Time kill curves for eugenol and antimicrobial drugs against pre-formed *C. albicans* (CAJ-01 and CAJ-12) and *S. mutans* MTCC497 biofilms. **a** CAJ-01, **b** CAJ-12, **c** *S. mutans* MTCC497

Dose dependent killing of pre-formed biofilms of *C. albicans* (CAJ-01 and CAJ-12) and *S. mutans* MTCC497 were observed at 2× SMIC of eugenol and antimicrobial drugs (fluconazole, amphotericin B and azithromycin). Treatment of pre-formed biofilms with 2× SMIC of eugenol showed strong fungicidal effect on *C. albicans* biofilms. Noticeable reduction in log_10_CFU count of *C. albicans* biofilm cells was observed within 12 h of treatment of eugenol. Whereas amphotericin B and fluconazole could not produce effective killing effect even upto 48 h of treatment (Fig. 3a, b).

Similarly, dose dependent killing effect was observed within 180 min after exposure with eugenol on pre-formed *S. mutans* MTCC497 biofilms. However, the effective biofilm eradication activity of azithromycin was noticed after 24 h of treatment on the pre-formed bacterial biofilms (Fig. 3c).

The interaction between euegnol and antimicrobial drugs (fluconazole, amphotericin B and azithromycin) was studied against the single and mixed biofilms and as depicted in Table 2. Synergy was observed between the eugenol and fluconazole against the CAJ-01 and CAJ-12 pre-formed biofilms with FICI value ranging from 0.156 to 0.250. However, indifferent interaction was noticed between eugenol and amphotericin B against the pre-formed *C. albicans* biofilm cells (FICI = 0.625). Similarly, synergistic interaction between eugenol and azithromycin against pre-formed *S. mutans* MTCC497 biofilms was recorded. Synergy was also noticed between eugenol and antimicrobial drugs (fluconazole and azithromycin) against the pre-formed *C. albicans* and *S. mutans* mixed biofilms. However, eugenol showed maximum synergy with azithromycin against *C. albicans* plus *S. mutans* MTCC497 mixed biofilms and FICI value was found 0.140. SMIC of azithromycin was reduced upto 8-folds against *S. mutans* MTCC497 in mixed biofilms.

**Table 2:** Combinational effects of eugenol and antimicrobial drugs on pre-formed *C. albicans* and *S. mutans* MTCC497 single and mixed biofilms

Test strains	Test combinations (concentration)			
1. CAJ-01	EUG/FLZ (µg/mL)		EUG/AMB (µg/mL)	
	EUG	FLZ	EUG	AMB
Alone MIC	400	512	400	256
Combination MIC	50	64	100	128
FICI	0.250		0.625	
Type of interaction	Synergy		Indifferent	
2. CAJ-12	EUG/FLZ (µg/mL)		EUG/AMB (µg/mL)	
	EUG	FLZ	EUG	AMB
Alone MIC	200	1024	200	32
Combination MIC	6.25	128	25	16
FICI	0.156		0.625	
Type of interaction	Synergy		Indifferent	
3. *S. mutans* MTCC497	EUG/AZI (µg/mL)			
	EUG	AZI		
Alone MIC	200	512		
Combination MIC	12.5	64		
FICI	0.187			
Type of interaction	Synergy			
4. CAJ-01 plus *S. mutans* MTCC497	EUG/FLZ (µg/mL)		EUG/AZI (µg/mL)	
	EUG	FLZ	EUG	AZI
Alone MIC	800	1024	800	1024
Combination MIC	25	128	12.5	128
FICI	0.156		0.140	
Type of interaction	Synergy		Synergy	
5. CAJ-12 plus *S. mutans* MTCC497	EUG/FLZ (µg/mL)		EUG/AZI (µg/mL)	
	EUG	FLZ	EUG	AZI
Alone MIC	200	2048	200	1024
Combination MIC	12.5	256	3.12	128
FICI	0.187		0.140	
Type of interaction	Synergy		Synergy	

*EUG* eugenol, *FLC* fluconaole, *AMB* amphotericin B, *AZI* azithjromycin

## Discussion

The inhibitory potential of antimicrobial agents was conducted on single biofilms and little work is being done so far under mixed condition. *C. albicans* and *S. mutans* are very deleterious in mixed biofilms. The interaction between these two pathogens produces resistant and recalcitrant infections in oral environment, which may further enhance complications in the treatment of oral infections (Peters et al. 2012; Gabrilska and Rumbaugh 2015). Therefore, the knowledge of mixed biofilms and their disruption strategy is utmost important to develop therapeutically useful approach. Due to increase in microbial resistance to existing antimicrobial drugs and decline in the formulation of new antimicrobial drugs, there has been witnessed an increased global interest in anti-infective natural products derived from medicinal plants. Various plant derived products (essential oils and phytocompounds) have so far been screened across the globe for their anti-biofilm activities. However, little attention was given to explore their therapeutic potential and its synergy with antimicrobial drugs against mixed biofilms.

Eugenol has potential to eradicate the single and mixed biofilms which are less susceptible to antimicrobial drug therapy. Emergence of phytocompounds resistant microbial strains is also not reported probably due to its multiple mode of action (Kavanaugh et al. 2012). The antimicrobial effect of eugenol has made them very effective alternative therapeutic agents.

In the present study, an effort has been made to evaluate eugenol potential to eradicate single and mixed biofilms alone and also in combination with known antimicrobial drugs against characterized *C. albicans* (CAJ-01 and CAJ-12) and *S. mutans* MTCC497 strains. This is a probably first attempt in this direction.

In this study, eugenol eradicates the mixed sessile cells of *C. albicans* and *S. mutans.* Interestingly, SMIC of eugenol is equivalent to PMIC of mixed *C. albicans* and *S. mutans* cells. This implies that eugenol under study are equally effective against both planktonic and sessile cells of *C. albicans* and *S. mutans.* Mixed biofilm cells of *C. albicans* plus *S. mutans* were sensitive to fluconazole in planktonic condition. In contrast, sessile cells were highly resistant to fluconazole in mixed condition. Apparently, SMIC of antimicrobial drugs (fluconazole and azithromycin) was increased upto1000-folds against *C. albicans* and *S. mutans* cells in the mixed bioiflms. Due to the limited penetration of antimicrobial drugs in these conditions, there is a need to increase the dose of the drugs which may further cause toxicological risks. It is also evident that eugenol was more effective on mixed species biofilm as compared to the single biofilm cells due to the increased susceptibility of mixed cells to eugenol (Jafri et al. 2019). Similar findings were also reported by the Fernandes et al. (2016). They studied the effect of farnesol on the pre-formed single and mixed biofilms of *C. albicans* and *S. mutans* and observed that farnesol was more effective on the mixed species biofilms compared to the single species biofilms.

The concentration dependent eradication of pre-formed single and mixed biofilms is also demonstrated by the viability assay. The findings of this study have revealed eradication of *Candida* and bacterial single biofilm cells occurred at higher MIC of eugenol. However, mixed biofilm cells were eradicated at MIC value of eugenol. This highlighted that there were lesser increase in tolerance of the eugenol towards the mixed bioflm cells compared to single biofilm cells.

Furthermore, efficacy of eugenol is demonstrated in terms of the time dependent killing of *C. albicans* and *S. mutans* matured biofilms. Eugenol showed efficacy within 12 h whereas antimicrobial drugs were ineffective even after 48 h of treatment against biofilm cells. Therefore, eugenol is considered as more cidal and potential antibiofilm agent compared to these antimicrobial drugs. Further, the eradication of pre-formed single and mixed biofilms at higher concentration of eugenol was also confirmed by scanning electron microscopy. Microscopy revealed distorted cell structure, reduced matrix production and eradication of single and mixed biofilm cells of *C. albicans* and *S. mutans* cells compared to untreated control at higher concentration of eugenol. Eradication of *Candida* biofilms in the presence of essential oils namely cinnnamaldehyde, linalool, *Melaleuca alternifolia, Mentha longifolia* is also reported by many workers de Campos Rasteiro et al. 2014; Serra et al. 2018; Tutar 2018).

In the light of synergistic approach, it is expected that synergy between eugenol and antimicrobial drugs against *C. albicans* and *S. mutans* could provide a new formulation for disease treatment (Nascimento et al. 2008; de Castro et al. 2015; Barbieri et al. 2017). Therefore, exploring nature of interaction between antimicrobial drugs with test compounds is pre-requisite to develop effective combinations. The nature of interactions can be indifferent, additive, synergistic and antagonistic. Synergy occurs when the combine effect of two test agents is greater than the effect of individual test agents (Cheesman et al. 2017). The mechanism of action in synergistic interaction may comprises of one or more actions like blocking of receptor, modification of the target site, degradation by enzymes, modification of the drugs, and accumulation of the antibiotics inside the microbes due to inhibition of outer membrane permeability (Bhardwaj et al. 2016; Stefanović, 2018; Ayaz et al. 2019).

In sessile mode of growth, eugenol has synergy with antimicrobial drugs (fluconazole and azithromycin) against the strains of *C. albicans* and *S. mutans* MTCC497. The synergistic interaction between eugenol and azithromycin has not been reported so far against *S. mutans*. Azithromycin inhibits the protein synthesis, quorum sensing and also biofilm formation (Parnham et al. 2014). Antibiotics in association with essential oils compounds can target multiple sites simultaneously and may reduce the drug related toxicity.

The FICI index study revealed the synergistic interaction between the eugenol and antimicrobial drugs to control the *C. albicans* and *S. mutans* single biofilms infections. This interaction studies were further exploited against the *C. albicans* and *S. mutans* mixed biofilms. This is a first report on the interaction study of eugenol with antimicrobial drugs against *C. albicans* and *S. mutans* mixed biofilms. In this study, eugenol showed maximum synergy with azithromycin against *C. albicans* and *S. mutans* MTCC497 in mixed biofilms. The SMIC of antimicrobial drugs were reduced upto 8-folds against the *Candida* and bacteria in mixed biofilms. The combination therapy has several advantages that may help to overcome the limitations of monotherapy for the treatment of mixed biofilms. The essential oil compound is equally effective against the bacterial and fungal cell which is not possible with antifungal and antibacterial drugs. The antifungal and antibacterial drugs will merely target the *Candida* and bacterial cells respectively. It is expected that eugenol perturbs the cell membrane integrity and allows the entry of drug into the microbial cell. This makes the antimicrobial drug available to the target site and resulted in improved efficacy.

Interestingly, the combination of antimicrobial drugs and eugenol could offers several advantages like enhanced potency, reduced dose of drugs, minimized toxicity which ultimately helps to inhibit or eradicate biofilms and overcome antimicrobial drug resistance (Chaouhan et al. 2017).

Thus, dose dependent killing effect of the eugenol-antimicrobial drug treatment suggests that these combinations could be subjected to treat the oral mixed biofilm infections.Further, the eugenol-drug combinations may be extended to different oral pathogens forming mixed pathogenic biofilms. Synergistic interaction between eugenol and antimicrobial drugs in planktonic and sessile mode needs to be evaluated on suitable animal model to assess its therapeutic efficacy. Further, based on the interaction of eugenol with antifungal and antibacterial drugs, a broad spectrum formulation may be standardized for topical application after assessing the toxicity issue if any.

Synergy of eugenol with azithromycin and fluconazole highlights the promising potential of phytocompounds to be used in combinational anti-infective therapy to combat single and mixed *C. albicans* and *S. mutans* biofilm associated infections. Further, in vivo efficacy is required for clinical application.
