# Correction to: Synergistic interaction of eugenol and antimicrobial drugs in eradication of single and mixed biofilms of *Candida albicans* and *Streptococcus mutans*

**DOI:** 10.1186/s13568-020-01149-6

**Published:** 2020-12-14

**Authors:** Huma Jafri, Gopa Banerjee, Mohd Sajjad Ahmad Khan, Iqbal Ahmad, Hussein Hasan Abulreesh, Abdulla Safar Althubiani

**Affiliations:** 1 Department of Agricultural Microbiology, Faculty of Agricultural Sciences, Aligarh Muslim University, Aligarh, 202002 India; 2 Department of Microbiology, King George Medical University, Lucknow, 226020 India; 3 Department of Basic Sciences, Deanship of Preparatory Year and Supporting Studies, Imam Abdulrahman Bin Faisal University, P.O. Box 1982, Dammam, 34212 Saudi Arabia; 4 Department of Biology, Faculty of Applied Science, Umm Al-Qura University, Makkah, Kingdom of Saudi Arabia

## Correction to: AMB Expr (2020) 10:185 https://doi.org/10.1186/s13568-020-01123-2

Following publication of the original article [1], the author noticed an error in the affiliation. The correct affiliation is given below:

Department of Basic Sciences, Deanship of Preparatory Year and Supporting Studies, Imam Abdulrahman Bin Faisal University, P.O. Box 1982, Dammam 34212, Saudi Arabia.

The original article has been corrected.
